# International experts’ perspectives on a curriculum for psychologists working in primary health care: implication for Indonesia

**DOI:** 10.1080/21642850.2014.929005

**Published:** 2014-07-15

**Authors:** Diana Setiyawati, Erminia Colucci, Grant Blashki, Ruth Wraith, Harry Minas

**Affiliations:** ^a^ Global and Cultural Mental Health Unit, Centre for Mental Health, Melbourne School of Population and Global Health, The University of Melbourne, Melbourne, Australia; ^b^ Faculty of Psychology, Universitas Gadjah Mada, Yogyakarta, Indonesia; ^c^ Nossal Institute for Global Health, The University of Melbourne, Melbourne, Australia

**Keywords:** primary mental health care, psychologist, Delphi methods, role, skill

## Abstract

Enhancing primary health care to incorporate mental health services is a key strategy for closing the treatment gap for people with mental disorders. The integration of psychological care into primary health care is a critical step in addressing poor access to mental health specialists. As the psychology profession is increasingly called upon to prepare psychologists for primary health care settings, an international experts' consensus is valuable in guiding the development of a high-quality curriculum for psychologists working in the primary health care context. A Delphi method was used to gain a consensus on the most appropriate roles and training for psychologists. Initial constructs and themes were derived from a detailed literature review and sent to 114 international experts in primary mental health care from five continents. Overall, 52 experts who participated agreed that psychologists should have wide-ranging roles and skills including clinical, health promotion and advocacy skills. This study has identified the specific roles and training needed by psychologists to enable them to work more effectively in primary health care settings. The consensus will inform the development of a curriculum for psychologists working in primary health care in Indonesia, and is part of a broader suite of studies.

## Introduction

A World Health Organisation (WHO) report, investing in mental health, found that more than 450 million people in the world suffer from mental health problems (2003). This makes mental illness one of the largest contributors to the global burden of disease. However, only a limited number of people with mental disorders are able to access appropriate treatment. In many countries, mental health is not included in any major health programme. In addition, mental health funding fares poorly compared with other public health challenges such as tuberculosis and human immunodeficiency virus (Patel, Koschorke, & Prince, 2011). A large gap exists between the burden of mental illness and the availability of services. At the global level, funding for mental health is less than two US dollars per capita per year on average. In high-income countries, the median of mental health expenditure reaches $44.84 per capita, 200 times higher than that in low-income countries where the median of mental health expenditure is just $0.20. Only 15 out of 39 low-income countries have mental health legislation, which barely covers 36% of the total population of those countries. Mental health legislation is a set of policies that may cover a broad range of issues in relation to mental health services, such as consent to treatment, human rights and consumer protection, as well as quality of services (World Health Organization, 2011).

Not only is funding inadequate in many countries, but there is also a fundamental misunderstanding about the sort of care that is needed to help people with mental health problems. General health professionals are often ill-equipped to handle common mental health problems, and a strong stigma attached to mental disorders persists both in the community and within the health professions (Epstein, Olsen, & Grey, 2012; Sartorius, 2011). Traditional models of care have also placed an overemphasis on hospital-based rather than community-based care for mental disorders (Patel et al., 2011).

Integrating mental health care into existing primary health-care services is considered as one promising solution to expanding access to mental health services (World Health Organisation, 2001). This view is based on the fact that mental disorders often initially present in primary health care, rather than in specialty mental health care. Many patients have comorbidity with physical health problems or indeed present to their general practitioner with physical symptoms rather than with a mental disorder per se (Hass & deGruy, 2004; Patel et al., 2011; Spitzer et al., 1995). Primary health care is a more affordable option than specialist care and thus is another way that treatment can be made more widely available (World Health Organisation & World Organisation of Family Doctor [WONCA], 2008).

Unfortunately, there are limitations to the level of care that existing primary health care professionals can provide. For example, a number of studies indicate that mental health problems are often under-diagnosed by general practitioners or primary health care providers (Connolly, Gaehl, Martin, Morris, & Purandare, 2011; Nuyen et al., 2005; Trude & Stoddard, 2003). The reasons for this may include a lack of training in mental health, as well as the workload and demands of primary health care. Therefore, great need still remains for specialist mental health professionals to support the integration of mental health services into primary health care (Hass & deGruy, 2004; Searight, 2010).

The integration of psychologists into primary health care systems is, therefore, one important step towards scaling up mental health services (Hass & deGruy, 2004). As an example, Moulding et al. (2007) reported good evidence for the effectiveness of psychotherapies delivered in primary health care. Indeed, psychotherapies delivered by psychologists had similar effects on the course of depression as that of antidepressant medication usually delivered by doctors (Hass, 2004). Elder and Silvers (2009) and Derksen (2009) also highlighted that the best way for psychologists to collaborate in primary care was to be co-located in primary health care clinics rather than in separate clinics. This approach also has the potential to reduce the stigma of mental illness and barriers to care.

Around the globe, the process of integration of psychologists into primary health care systems itself is a gradually evolving process. Two studies in the Netherlands and the USA showed that it took several years to initiate this type of reform within the existing systems (Derksen, 2009; Elder & Silvers, 2009). Potential barriers to be considered in the process of integration include clinical, operational and financial barriers, as well as the training required for psychologists working in medical or primary health care settings (Gunn & Blount, 2009).

In Indonesia, the integration of psychologists into primary health care commenced in 2004. It was initiated by the Sleman District Health Office (a district within Yogyakarta Province) in collaboration with the Faculty of Psychology, Universitas Gadjah Mada (Retnowati, 2011). Similar to the situation in other countries (Hass & deGruy, 2004), the current training for psychologists does not specifically prepare them for work in primary health care settings.

The objective of this study is to reach a consensus among experts from around the world based on their own experiences and perspectives about the key roles and training required for psychologists who will be working in primary health care. This consensus will inform the development of a curriculum for psychologists working in primary health care in Indonesia, and is part of a broader suite of studies (Setiyawati, Blashki, Wraith, Colucci, & Minas, 2013a, Setiyawati, Blashki, Wraith, Colucci, & Minas, 2013b, Setiyawati, Blashki, Wraith, Colucci, & Minas, 2014).

## Method

### Delphi method

The Delphi method is one of the most commonly used consensus research methods and has been used extensively in health research since the mid-1970s (Boulkedid, Abdoul, Loustau, Sibony, & Alberti, 2011; De Villiers, De Villiers, & Kent, 2005). In mental health research, for example, it has been successfully used to develop a research agenda for refugee mental health and guidelines for suicide prevention in Asian countries and to improve the cultural responsiveness of mental health services (Colucci, Kelly, Minas, Jorm, & Chatterjee, 2010; Colucci, Kelly, Minas, Jorm, & Nadera, 2010; Colucci, Kelly, Minas, Jorm, & Suzuki, 2011). This method allows a panel of experts to achieve consensus about specific issues without meeting each other (Turoff & Linstone, 2002).

### Procedure

This study utilised a Delphi method with questionnaires to collect international expert opinions. The methodology of this research consisted of four main phases:

1. a literature review of the roles and training of psychologists working in primary health care;
2. development of a questionnaire based on the literature review;
3. formation of a panel of international experts in primary mental health care;
4. delivery of online surveys, including a background information survey using the Delphi method (Turoff & Linstone, 2002), to seek consensus around a series of statements regarding key aspects of the roles and training for psychologists in primary health care.

#### (1) Literature review

The questionnaire was developed on the basis of literature published between 1995 and 2010 (the beginning of this study) regarding psychologists working in primary health care. In 1995, the Committee for the Advancement of Professional Psychology (CAPP) of the American Psychological Association (APA) appointed an eight-member taskforce to review opportunities and obstacles for psychologists working in primary health care. Therefore, the year 1995 was used as the starting point of this review as this marked a significant shift of focus towards the integration of psychology into primary health care (Bray, 1996).

The literature has been explored systematically through databases, namely PsycINFO, Science Direct, Medline, Expanded Academic ASAP (Gale), JSTOR, APAFT: Australian Public Affairs, Web of Science (ISI), EBSCO, Informit and University of Melbourne Digital Repository (DigiTool). The keywords used for the search were ‘psychologist AND primary care’; ‘psychologist AND primary health care’; ‘mental health AND primary care’; ‘mental health AND primary health care’ and ‘primary care psychology OR primary health care psychologist’. A daily alert for new literature was set up from these databases.

#### (2) Questionnaire development

Based on a review of the literature, a list of 120 items about roles and skills of psychologists working in primary health care was generated for the questionnaire. The items were organised based on Talens, Fraser and Cauley's three-dimensional cube concept about roles and skills, and divided into five dimensions: (1) in relation to patients; (2) in relation to other primary health care psychologists; (3) in relation to primary health care as an organisation/service provider; (4) in relation to community and (5) in relation to policy (Talen, Fraser, & Cauley, 2005). The items identified in the literature review constituted the substrate on which the survey for the Delphi method was developed.

#### (3) Panel formation

As one of the quality indicators of the Delphi method is the heterogeneity of experts, the researcher invited experts in primary mental health care across different continents and from various professions to form the panel (Boulkedid et al., 2011). The experts were individually invited based on the following inclusion criteria: working in mental health service area for at least four years; holding a significant position in a primary health care psychology and a representative from one of the countries that have a programme of integration of psychology into primary health care (e.g. USA, Norway, UK, Canada, Netherlands, Iran, Sri Lanka, Germany, Australia and Thailand).

Potential participants were identified by reviewing their scientific publications on this topic, as well through suggestion by other participants or key professional organisations, such as the Australian Psychological Society (APS), APA, and the WHO. The number of panel members in previous Delphi studies has varied considerably from 15 to 60 (Hasson, Keeney, & McKenna, 2000). A panel usually consists of 15–30 participants from the same discipline, or 5–10 per category if they are drawn from different professional groupings (De Villiers et al., 2005). As the aim of this research was to build a panel that represented the opinions of experts from different continents and regions, 114 experts were invited to participate in the Delphi process.

#### (4) Delivery of the online survey

The potential participants identified and recruited to form the expert group were sent a personalised link to an online Delphi survey hosted on the SurveyMonkey website. They were also given the option to receive the paper version of the survey by mail if they wished, to facilitate the participation of experts with limited Internet access.

The due date to complete the survey was two weeks from the date of the invitation. Four days before the due date, the first reminder email was sent to participants who had not yet responded. The second reminder email was sent to any participants who had still not yet responded on the day after the due date, and they were informed that the due date was extended for the next nine days. The last remainder email to participants who had not responded was sent two days before the final due date.

Participants were provided with a plain language statement and informed consent form when they accessed the personalised link. If they agreed to continue and clicked ‘yes’, they were led to the questionnaire. The first round Delphi survey included a total of 120 items, which were divided into the domains listed in Table 1, and are based on Talen et al. (2005). The participants were asked to record their opinion by clicking a radio button for the answer they chose, selecting from a drop-down menu that contained multiple-choice options or adding an additional comment on the webpage.

**Table 1.** Number of items in the questionnaire based on each domain.

Domain	Roles (numberof items)	Skills (number of items)
1. In relation to patients	12	26
		28 (counselling skills)
		9 (therapeutic methods)
2. In relation to other primary health care providers	5	12
3. In relation to primary health careas organisation/service provider	3	10
4. In relation to community	3	4
5. In relation to policy	3	5

**Figure 1.**
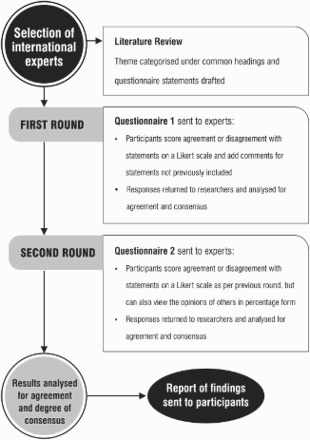
The flow chart of Delphi process.

For Round 1, surveys were divided into two sections. Firstly, participants were asked to provide some background information, including their profession, age, sex, country of service and length of service in work relevant to primary mental health care or mental health system development. Secondly, participants were invited to agree or disagree with a list of items (statements) based on a continuum of responses: strongly agree, agree, disagree or strongly disagree. Each item was compulsory (i.e. no question could be skipped). At the end of each section, participants were also invited to add comments and suggest any additional item that was not indicated in the list. The criteria in Table 2 were chosen in order to determine which statements in the questionnaire had reached consensus. As in previous studies (Colucci et al., 2011), the results were collected and feedback was provided to participants for use in the next round (see the flow chart in Figure 1).

**Table 2.** Criteria to determine the level of agreement.

Statement's status	Criteria
Endorsed	Rated by 80% or more of participants as strongly agree/agree
• as key aspects of the roles or skills of psychologists working in primary health care	
Re-rated	Rated by between 70% and 79% of participants as strongly agree/agree
• designated to be re-rated by participants in the second round Delphi survey	
Rejected	Rated by less than 70% of participants as strongly agree/agree

In Round 2, eight statements that were rated as strongly agree/disagree by between 70% and 79% of participants were re-rated. The results of the first round were sent back to participants so they could re-rate these statements in light of other participants' opinions. Thirty-eight new statements were also generated from participants' additional comments from Round 1. Participants were asked to record their agreement/disagreement with these new statements. At the end of Round 2, participants were asked to give their opinion on field placement in primary health care, the appropriate length of field placement and what should be included in the field placement.

## Results

### Round 1

Of the 114 experts who were invited to take part in this study, 52 (46.0%) completed the Round 1 questionnaire. More than one quarter were from the American Continent (29.0%), followed by experts from Australia (21.0%), Asia (19.0%) and Europe (17.0%). Less than 10% of experts came from Africa (6%), and four experts working internationally (8.0%) did not identify as being based in any specific country or continent. A variety of professions were also represented in the composition of participants. Almost half of the participants were psychologists (48.1%), and less than 20% were psychiatrists (17.3%) or medical doctors (15.4%). Other professions constituted less than 10% of participants: lecturer (9.6%), policy-maker (7.7%), social worker (3.8%), clinician (3.8%) and community mental health nurse (1.9%) (Table 3).

**Table 3.** Socio-demographic characteristics of participants.

Attribute	Frequency	Percentage^a^
*Location of workplace*		
Africa	3	6
America	15	29
Australia	11	21
Europe	9	17
Asia	10	19
International	4	8
*Gender*		
Male	25	48.1
Female	27	51.9
*Profession*		
Psychologist	25	48.1
Psychiatrist	9	17.3
Medical doctor	8	15.4
Community mental health nurse	1	1.9
Social worker	2	3.8
Clinician	2	3.8
Policy-maker	4	7.7
Lecturer	5	9.6
Others	10	19.2
*Publication in primary mental health care*		
Yes	42	82.4
No	9	17.6

^a^The sum of the percentage is more than 100, because participants chose more than one profession.

One hundred and six of the original statements (88%) achieved agreement and were endorsed to be key roles or skills for a psychologist working in primary health care. Six items reached an agreement rate lower than 70%, so were rejected. The eight items that reached between 70% and 79% agreement were sent through to the second round. The participants were invited to re-rate these eight items in light of other experts' opinions from Round 1. Thirty-eight new items generated from participants' comments in Round 1 were also sent to be rated by participants in Round 2. The new items generated from participants' comments covered the five domains as previously established in the first round.

### Round 2

The 52 experts who participated in Round 1 were invited to take part in Round 2, and 41 of them (79%) completed the Round 2 questionnaire. Thirty-six of the 38 items suggested at Round 1 reached consensus (i.e. were rated as strongly agree/agree by 80% or more of the experts). Only two items that were generated from the suggestions provided in Round 1 achieved a rate of agreement below 80%. All eight items that were re-rated reached a level of agreement higher than 80%. Therefore, because there was no significant disagreement, the Delphi process was closed after this round.

#### Endorsed statement

The result from the two Delphi rounds was 150 items that were rated as agree/strongly agree by 80% or more of the panel members. The complete list of endorsed statements is available upon request. Table 4 lists some examples of endorsed statements for psychologists' roles and Table 5 lists some examples of endorsed statements for psychologists' skills.

**Table 4.** Examples of endorsed statements (psychologists’ roles).

Endorsed statement	Mean	Consensus level (%)
In relation to *patients*, psychologists’ role/s should include:		
1. providing psychological treatment	4.73	96.10
2. addressing psychological aspects of patients’ illnesses	4.71	96.10
3. conducting assessments	4.65	96.20
In relation to *other primary care providers*, psychologists’ role/s should include:		
1. referring patients when necessary	4.65	96.20
2. accepting referrals from other health care providers	4.65	96.20
3. providing secondary consultation about patients to other primary care providers	4.62	96.20
In relation to *primary care as an organisation/service provider*, psychologists' role/s should include:		
1. programme development	4.44	92.30
2. conducting evaluation of services	4.36	90.40
3. conducting research	4.3	86.50
In relation to *community*, psychologists’ role/s should include:		
1. promoting mental health in the community	4.46	92.30
2. providing education about mental health	4.4	88.50
3. promoting understanding of psychologists’ role in the community	4.3	82.70
In relation to *policy*, psychologists’ role/s should include:		
1. advocating community focus in mental health policy	4.44	92.30
2. interacting with non-health sector (e.g. education and employment) regarding mental health issues	4.4	94.30
3. advocating mental health in public policy	4.38	94.30

**Table 5.** Examples of endorsed statements (psychologists’ skills).

Endorsed statement	Mean	Consensus level (%)
In relation to *patients*, psychologists should have *skills/training* in the following areas:		
1. working with families in primary care	4.63	100.00
2. evidence-based therapies	4.61	100.00
3. prevention of common mental health problems seen in primary care	4.59	96.00
In relation to *other primary care providers*, psychologists should have *skills/training* in the following areas:		
1. working collaboratively with other health professionals	4.8	96.10
2. bio-psychosocial model	4.67	96.10
3. help match patients’ need for treatment with expertise of primary care providers	4.59	98.10
In relation to *primary care as an organisation/service provider*, psychologists should have *skills/training* in the following area:		
1. ethical issues in primary care	4.59	96.00
2. primary care's culture and language	4.49	96.00
3. professional issues in primary care	4.45	96.10
In relation to *community*, psychologists should have the *skills/training* in the following areas:		
1. understanding multicultural diversity	4.47	100.00
2. understanding community resources	4.25	98.00
3. understanding local concerns about stigma	4.2	98.00
In relation to *policy*, psychologists should have *skills/training* in the following areas:		
1. understanding mental health policy	4.71	100.00
2. understanding health care systems	4.51	96.10
3. understanding general health policy	4.49	96.00

Additional comments from participants were analysed and matched with existing statements listed in Round 1. Any comments that identified new ideas were generated into new statements. Table 6 provides examples of the comments and suggestions that led to the generation of additional statements (quoted verbatim).

**Table 6.** Examples of statements generated from comments (Round 2).

No.	Comments	Statements
1	‘Apart from above role, in primary health care, a psychologist also has to play an important role in dealing with the social wellbeing of the patients, i.e. helping the immediate family members to maintain a healthy environment at home’.	In relation to patients, psychologists’ role/s should include dealing with the social well-being of patients.
2	‘Educating primary health care providers regarding the signs of common psychological problems like depression and substance abuse’.	In relation to other primary health care providers, psychologists’ role/s should include educating primary health care providers regarding the signs of common psychological problems.
3	‘It is very important to have relationships with respected community figures/leaders, so that the patient population “buys in” to seeing a mental health provider’.	In relation to community, psychologists’ role/s should include building relationships with respected community figures/leaders.

Comments were not generated into new statements if they did not identify anything new or different from existing statements or if they did not meet the criteria to be added as a new statement (e.g. if they did not suggest a clear point). Table 7 lists examples of comments and suggestions that were ultimately not used to generate a new statement (quoted verbatim with minor editing). The additional file for complete endorsed items, including percentage of panel member agreement, is available on request.

**Table 7.** Examples of comments that were not generated into new statements.

No.	Comments	Notes
1	‘Depending the setting and area of implementation tasks of psychologists might be limited or expanded’.	Unclear suggestion
2	‘Again it is optimal that the primary psychologist be involved in these roles-however, time constrains often mitigate against it’.	Unclear suggestion
3	‘Need an understanding of all and at least one approach developed more fully, probably CBT as biggest evidence based’.	The idea of this statement had been presented in the existing survey: ‘In relation to patients, psychologists should have the skill/training in cognitive behaviour therapy’.
4	‘In reference to contributing to the primary health care team development. I think this can be an important role for psychologists for a team of which they are not a member. For the team on which the psychologist is a member, the psychologist may very well bring skills to the team that help to facilitate team development’.	The idea of this statement had been presented in the existing survey: ‘In relation to patients, psychologists’ role/s should include contributing to the primary health care team development’.

Overall, in regard to the role of psychologists in relation to patients, there was a strong consensus that psychologists in primary health care ought to be providing psychological treatments, addressing psychological aspects of patients' illness and conducting assessments. In line with these roles, the training/skills for which there was the strongest consensus were working with families in primary health care, providing evidence-based therapies, preventing mental illness and providing psycho-education.

Furthermore, in regard to the role of psychologists in relation to patients, there was also strong consensus that psychologists in primary health care ought to be well trained in counselling skills, including listening skills, verbal communication, assessing risk of harm to oneself and others and understanding the patients' points of view. The therapeutic methods that were most consistently agreed upon as important for psychologists were cognitive behavioural therapy and individual psychotherapy including brief therapy. Counselling approaches, such as giving immediate and concrete solutions in a short session of consultation, did not achieve a strong level of consensus among the experts.

Regarding the roles of psychologists in relation to other primary health care providers, there was strong agreement that psychologists ought to educate other health care providers about matters pertaining to psychology and about the signs of common psychological problems. There was also the idea that psychologists should be prepared to refer when necessary. In line with these findings, there was a high agreement on the type of skills and training needed to work collaboratively with other health professionals, such as training to understand the bio-psychosocial model, skill to share information and to match patients' needs for treatment with the expertise of primary health care providers.

Regarding the roles of psychologists in relation to primary health care as an organisation/service provider, most of the experts agreed that important areas for psychologists to be involved in ought to include contributing to quality improvement of primary health care, promoting evidence-based practice, and programme development. Developing standard mental health screening instruments and continuous monitoring of programmes are examples of quality improvement activities given by participants. Another participant mentioned getting involved in any health promotion activities of the organisation as an example of programme development. The items with the highest rates of consensus were to train psychologists to understand different professional cultures, ethical issues in primary health care and the culture and language primary health care.

In relation to the community, the roles of psychologists in primary health care that reached the highest rates of consensus were promoting mental well-being, understanding of the relationship between behaviour and physical health and stigma reduction. In line with these results, understanding multicultural diversity, community resources and local concerns about stigma were training issues with the highest consensus.

In relation to policy, psychologists' roles for which there were highest rates of consensus were advocating for mental health in primary health care in public policy, advocating for a community focus in mental health policy and interacting with the non-health sector regarding mental health issues. The training regarding understanding mental health policy, health care systems and general health policy also achieved high rates of consensus.

Regarding a field placement in primary health care, 95% of the experts agreed that this placement is needed as part of preparing psychologists to work in primary health care. The recommended duration required for the placement varied from six weeks to two years.

#### Rejected statements

Only eight items from the two Delphi rounds were rejected because they were rated as agree/strongly agree by less than 70% in Round 1 or less than 80% of the panel members in Round 2. The items that were rated as agree/strongly agree by between 70% and 80% of experts in Round 1 were re-rated by the experts in Round 2. Then, the items that were rated as agree/strongly agree by between 70% and 80% of experts in Round 2 were not sent back to the experts. It was due to insignificant numbers of disagreement to make the questionnaire for the third round. Therefore, the items that were rated as agree/strongly agree by less than 70% of panel members in Round 1 or less than 80% of panel members in Round 2 were rejected. The eight rejected items are listed in Table 8.

**Table 8.** Rejected statements.

No.	Statement	Consensus level (%)
1	In relation to patients, psychologists’ role/s should include providing prescriptions for psychotropic medications	15.4
2	In relation to patients, psychologists’ role/s should include conducting home visits	61.6
3	In relation to patients, psychologists should have skills/training in prevention of common chronic illness seen in primary care (e.g. hypertension and diabetes)	68.6
4	In relation to patients, psychologists should have skills/training in psychotropic medication	64.7
5	In relation to patients, psychologists should have counselling skills/training, including giving immediate and concrete solutions in a short session of consultation	62.8
6	In relation to patients, psychologists should have skills/training in pharmacotherapy	36.4
7	In relation to patients, psychologists should have skills/training in spiritual/religious aspects in prevention or mental health treatment	70.7
8	In relation to primary care as an organisation/service provider, psychologists should have skills/training in action research	68.3

Overall, seven of the eight rejected statements were in relation to patients and the rest is in relation to primary health care as service providers. The item related to expertise in action research specifically did not achieve a high rate of consensus among the participants as a skill needed by psychologists working as service providers in primary health care.

In relations to patients, the topic with the least consensus related to the role of psychologists in prescribing psychotropic medications. Consistently, there was little consensus surrounding the training of psychologists about psychotropic medication.

The other role in relation to patients that attracted a wide range of views and did not reach consensus was conducting home visits. Training in prevention of common chronic illnesses seen in primary health care (e.g. hypertension and diabetes), and spiritual/religious aspects in prevention or mental health treatment also did not reach consensus. Counselling approaches, such as giving immediate and concrete solutions in a short session of consultation, also did not achieve a strong level of consensus among the experts.

## Discussion

The main finding of this research is that psychologists should have wide-ranging roles and skills when working in primary health care, including clinical, health promotion and advocacy skills. This study has identified the specific roles and training needed by psychologists in relation to patients, in relation to other primary health care providers, in relation to primary health care as an organisation/service provider, in relation to community and in relation to policy. These roles and skills will enable them to work more effectively in primary health care settings.

The results of this research regarding psychologists' roles and skills in relation to patients are in line with other curricula undergoing development, such as the postdoctoral training programme in psychology developed in Michigan (Vogel, Kirkpatrick, Collings, Cederna-Meko, & Grey, 2012). Another example is the training curriculum that had been developed by the faculty at Wright State University's School of Professional Psychology for more than 10 years (Talen et al., 2005).

Interestingly, the topics with least consensus in the current research related to the question of the prescription of psychotropic medications by psychologists. This lack of agreement is in line with the debate in the literature about the prescribing rights of psychologists (Johnson, 2009; Newman, 2013; Rae, Jensen-Doss, Bowden, Mendoza, & Banda, 2008).

Although training for psychologists working in primary health care is still under development, there is already an existing body of literature in this area (Bray, 2004; Hass, 2004; McDaniel, Belar, Schroeder, & Hargrove, 2002; McDaniel, Hargrove, Belar, Schroeder, & Freeman, 2004; Searight, 2010; Talen et al., 2005). Most of the results of the current study confirmed the points highlighted by this existing literature. However, this was the first international study in this area where findings were determined through the Delphi consensus method.

This study has demonstrated that there are similarities among experts from all over the world in their perceptions of the roles and skills of psychologists working in primary health care. It is possible to reach consensus on this topic between experts who come from various cultures and experiences. The Delphi method has been successfully used in the education area for developing curricula, as well as for designing innovative programmes in teaching (Kizawa et al., 2012; Rayfield, Murphy, Briers, & Lewis, 2012). In the mental health area, the Delphi method has been employed to develop guidelines and protocols (Colucci et al., 2011; Kelly, Jorm, & Kitchener, 2009; Kelly, Jorm, Kitchener, & Langlands, 2008; Maarsingh et al., 2009; Perry et al., 2010). The Delphi method has been endorsed for use in developing countries that usually have limited research evidence (Minas & Jorm, 2010).

This research investigated international experts' opinions in order to reach a consensus. These opinions are based primarily upon the experts' experiences in their native countries; therefore, how these opinions are applied to a developing country such as Indonesia needs careful consideration. Based on others' experiences in various countries, a developing country like Indonesia can learn about challenges, priorities and management during the initial stages of the process of integration of psychologists into primary health care.

Direct comparison between the international and Indonesian contexts, however, is not always the best way to learn or transfer knowledge from one area to another. The ideas of Western mental health have a global influence, not only upon the diagnosis and treatment of mental health illness, but also upon the meaning of mental health itself. According to Watters (2010), Western ideas of mental health can often make cultural assumptions and tend to bring a uniform, homogenised approach to mental health treatment. Sethi (2013) pointed out that the expression of mental illness across cultures varies. The diversity of manifestations of mental illness is starting to be discussed also in Indonesia, where people more commonly make physical complaints, such as ‘headache’, ‘hot chest’, ‘uncomfortable stomach’ or ‘hearing problem’, rather than expressing illness in terms of depression or anxiety (Retnowati, 2011). Therefore, in order to apply lessons from other countries and cultures to the Indonesian context, great care needs to be taken to consider the voice of Indonesian stakeholders and their cultural context. For this reason, in order to develop a curriculum for psychologists working in primary health care in Indonesia, this study has been followed by a number of other studies, which include interviews with Australian and Indonesian experts. Furthermore, another Delphi study for Indonesian experts will be reported separately.

A limitation of this research is the unequal distribution of participants from all around the world. The dominant proportions of Western experts are from America, Australia and Europe, and there are limited numbers of Asian and African participants. Significant effort was made to recruit an equal proportion of experts from all regions, but due to a limited network of contacts, and the limited number of publications from experts, in this field from Asia and Africa, it was difficult to get a higher proportion of experts from these continents.

This research can act as a foundation for future research into the integration of psychologists into primary health care. The evaluation of mental health services provided in primary health care is one important research aspect that needs to be conducted to monitor the impact of this integration. Cultural differences regarding perceptions of mental health treatments, including the application of Western psychotherapy techniques within non-Western countries, are another important research area that needs to be investigated in order to make future recommendations regarding Indonesian mental health care. This research can also serve as a background for human resource development research for developing psychology as a new workforce in the primary health care area.

## Conclusion

This is the first international consensus achieved through a Delphi study in the area of developing curriculum for psychologists working in primary health care. The representativeness of the experts from all over the world was carefully considered. The results of this study confirm the findings of the essential literature in this area. This study has identified the specific roles and training needed by psychologists to be able to work effectively in primary health care settings. This finding can inform curriculum development for psychologists working in primary health care.
